# Long noncoding RNAs: pivotal regulators in acute myeloid leukemia

**DOI:** 10.1186/s40164-016-0059-9

**Published:** 2016-12-12

**Authors:** Shuyong Wei, Kankan Wang

**Affiliations:** 1 State Key Laboratory of Medical Genomics and Shanghai Institute of Hematology, Ruijin Hospital, Shanghai Jiao Tong University School of Medicine, 197 Ruijin Er Rd, Shanghai, 200025 China; 2 Sino-French Research Center for Life Sciences and Genomics, Ruijin Hospital, Shanghai Jiao Tong University School of Medicine, Shanghai, 200025 China

**Keywords:** Long noncoding RNAs, Acute myeloid leukemia, Myelopoiesis

## Abstract

Long noncoding RNAs (lncRNAs) have emerged as a class of pivotal regulators of gene expression. Recent studies have shown that lncRNAs contribute to the initiation, maintenance, and development of acute myeloid leukemia (AML). In this review, we summarize the current knowledge of the lncRNAs that play critical roles in AML. We first briefly describe the characteristics of lncRNAs, and then focus on their regulatory roles in AML, including the modulation of differentiation, proliferation, cell cycle, and apoptosis. We further emphasize the action of lncRNAs during leukemogenesis by describing how they interact with RNA, protein and chromatin DNA to exert their functions. We also highlight an urgent need to investigate the mechanisms by which lncRNAs contribute to the pathogenesis of AML. Finally, we discuss the prognostic value of lncRNAs in AML patients.

## Background

Acute myeloid leukemia (AML) is a group of hematopoietic malignancies with various genetic abnormalities, including chromosomal translocations and/or somatic mutations, which are mainly responsible for the abnormal proliferation, differentiation or survival of myeloid progenitors [1, 2]. Extensive studies have established the regulatory roles of protein-coding genes in the initiation, maintenance, and development of AML, which constitutes our main knowledge of the pathogenesis of AML.

Recently, long noncoding RNAs (lncRNAs) have emerged as a novel class of pivotal regulators of gene expression and has received increasing attention in the field of AML. LncRNAs are operationally defined as RNA larger than 200 base pairs that appear to lack coding potential. They participate in various cellular processes, like inflammatory response [3], neuronal activity [4] and erythropoiesis [5]. Mechanistically, lncRNAs could promote the strength of specific enhancer-promoter looping and thus contribute to gene activation [6–8], regulate protein modifications and activities, sequester microRNAs, and serve as precursors of small RNAs [9–11]. Based on the genomic locations where lncRNAs are transcribed, they can be classified into the following groups: (1) sense, which overlap with at least part of another gene in the same strand; (2) antisense, which overlap with at least part of another gene on the opposite strand; (3) intronic, which originate from the intron of another gene; (4) intergenic, which does not overlap with any gene. In addition to the above four classes, it is possible to characterize an additional fifth category: (5) chimeric, which are the fusion products due to chromosomal rearrangements, based on a study showing the existence of fusion transcripts between protein-coding genes and lncRNAs in AML [12]. Since only two known protein-lncRNA fusions have been identified in AML and their function has not been elucidated [12], the further investigation would be required. In this review, we summarize the recent progress in the knowledge of lncRNAs in AML, including their biological functions, the mechanisms behind their actions, the upstream regulation and the prognostic values in the clinic.

## The roles of lncRNAs in myeloid leukemia

The latest studies have demonstrated that lncRNAs contribute to many critical signaling pathways in AML development and therapy. We summarize the reported AML-related lncRNAs and their roles in Table 1.

**Table 1:** LncRNAs in AML

LncRNAs	Classification	Function	Target genes	Reference
RUNXOR	Sense	Be involved in chromosomal translocation	RUNX1	[13]
HOTAIRM1	Antisense	Regulate myeloid differentiation and cell cycle enhance the autophagy pathway regulates chromatin state and architecture	HOXA1, HOXA4, CD11b and CD18 miR-20a/106b and miR-125b	[14–17]
HOXA-AS2	Antisense	Act as an apoptosis repressor	Unknown	[18]
PU.1-AS	Antisense	Inhibit the translation of PU.1	PU.1	[19]
WT1-AS	Antisense	Control WT1 expression	WT1	[20]
EGO	Intronic	Regulate MBP and EDN expression	MBP and EDN	[22]
IRAIN	Intronic	Be engaged in long-range intra chromosomal interactions	IGF1R	[23]
BGL3	Intergenic	Sensitize leukemic cells to undergo apoptosis	miR-17, miR-93, miR-20a, miR-20b, miR-106a and miR-106b	[24]
CCAT1	Intergenic	Repress monocytic differentiation and promote cell growth	miR-155	[25]
CCDC26	Intergenic	Control the growth of AML cells	c-Kit	[26]
HOTAIR	Intergenic	Induce cell growth and inhibit apoptosis	miR-193a and c-Kit	[27]
NEAT1	Intergenic	Regulate ATRA-induced myeloid differentiation	Unknown in AML	[28]
PVT1	Intergenic	Regulate proliferation of promyelocytes	MYC	[21]
UCA1	Intergenic	Sustain proliferation of AML cells	p27kip1	[29]
PVT1-NSMCE2	Fusion	Unknown	Unknown	[12]
BF104016-NSMCE2	Fusion	Unknown	Unknown	[12]

LncRNAs exert important roles in myeloid differentiation and can respond to differentiation induction therapy. At present, two lncRNAs, HOTAIRM1 and NEAT1 are known to regulate the differentiation of AML cells. HOTAIRM1 is a myeloid-specific long non-coding transcript, which is transcribed from the locus between HOXA1 and HOXA2 genes. HOTAIRM1 regulates myeloid differentiation genes such as CD11b and CD18, and its knockdown impairs all-trans retinoic acid (ATRA)-induced granulocytic differentiation [14]. This work could serve as a paradigm for exploring lncRNAs in AML, from the discovery of the lncRNA candidate to the investigation of the biological function. This work also has an interesting finding—HOTAIRM1 is derived from HOXA clusters, and in turn regulates the nearby genes in HOXA cluster. Whether this regulation is direct or indirect awaits further investigation. Another example is NEAT1, a widespread and abundant long noncoding RNA. Although myeloid differentiation is usually considered to be dominantly controlled by highly myeloid-specific factors, NEAT1 has been reported to be responsive to ATRA and be indispensable for ATRA-mediated myeloid differentiation [28]. This observation indicates that myeloid differentiation also requires commonly expressed long noncoding transcripts. However, since both studies of HOTAIRM1 and NEAT1 mentioned above were conducted in myeloid leukemia cells, it has yet to be determined whether HOTAIRM1 and NEAT1 are required for normal myelopoiesis. Further in vivo studies are needed to evaluate their roles in normal hematopoiesis and the development of AML.

LncRNAs also exert effects on proliferation, cell cycle and apoptosis in AML cells. Such lncRNAs tend to be expressed more widely than those myeloid-specific transcripts that regulate differentiation. A typical example is lncRNA PVT1, which can promote the proliferation of AML cells [21]. The oncogenic activity of PVT1 is closely related to MYC, whose overexpression can lead to hyper proliferation of cancer cells. A gain-of-function of both the MYC gene and the PVT1 lncRNA due to the amplification of 8q24.21 is observed in about 10% of AML patients [30]. On the one hand, PVT1 protects MYC from degradation by direct physical interaction [30]. On the other hand, PVT1 acts as a microRNA precursor for indirect regulation of MYC [31]. The PVT1 locus can produce six annotated oncogenic microRNAs [31, 32], and one of these microRNAs, hsa-miR-1204, which is derived from the exon 1b, has been reported to be able to enhance the expression level of MYC [31]. From the example of PVT1, we should note that lncRNAs may have multifaceted roles and can function through multiple ways. Also, the co-amplification of PVT1 and MYC informs us that it might be interesting to see the association or causal relationship of copy number of lncRNAs with leukemogensis. In addition to PVT1, lncRNA UCA1 has also been reported to have the capability to modulate the proliferation of AML cells [29]. UCA1 silencing by short-hairpin RNA transduction results in a significant reduction of cell proliferation, with an increase in the G1 phase and a decrease in the S phase. Another example is lncRNA CCAT1, which can coordinate the proliferation and differentiation of AML cells [25]. Overexpression and knockdown experiments demonstrate that CCAT1 inhibits the PMA-induced monocytic differentiation as well as promotes the proliferation of AML-derived HL60 cells [25]. As to apoptosis of AML cells, Xing et al. [27] found that HOTAIR knockdown inhibits cell growth and colony formation, and also induces the apoptosis of AML cells [27]. Of note, the oncogenic properties of these lncRNAs are not limited to leukemia, but can also be observed in solid tumors. For example, PVT1 can promote the proliferation of hepatocellular carcinoma and non-small cell lung cancer cells [33, 34]. Similarly, CCAT1 can promote the proliferation and invasion of colon cancer cells, gallbladder cancer cells and hepatocellular carcinoma cells [25, 35, 36]. These observations indicate that such lncRNAs usually share similar functions in leukemia and other types of malignancies. Further experiments are required to investigate the underlying mechanism by which these lncRNAs control the cellular phenotypes—proliferation, cell cycle and apoptosis in AML cells.

Recently, the discovery of a novel class of lncRNAs piques the curiosity of scientists and potentially ignites their efforts to characterize them: fusion transcripts between coding genes and lncRNAs. In an AML patient sample and an AML-derived HL-60 cell line, it is verified that NSMCE2 rearrangement gives rise to two novel chimeric genes, PVT1-NSMCE2 and CCDC26-NSMCE2 [12]. Although their identities have been revealed, their involvement in leukemogenesis is elusive to date. It is likely that PVT1-NSMCE2 and CCDC26-NSMCE2 may contribute to the leukemogenesis through the oncogenic activity of PVT1 and CCDC26 [26]. At present, our understanding of the fusion transcripts between lncRNAs and protein-coding genes is still in its infancy. Further experiments are required to determine the causal relationship between the protein-lncRNA-fusion transcripts and leukemia as well as the mechanism behind their behaviors.

## Mechanisms of lncRNA action in acute myeloid leukemia

**Fig. 1:**
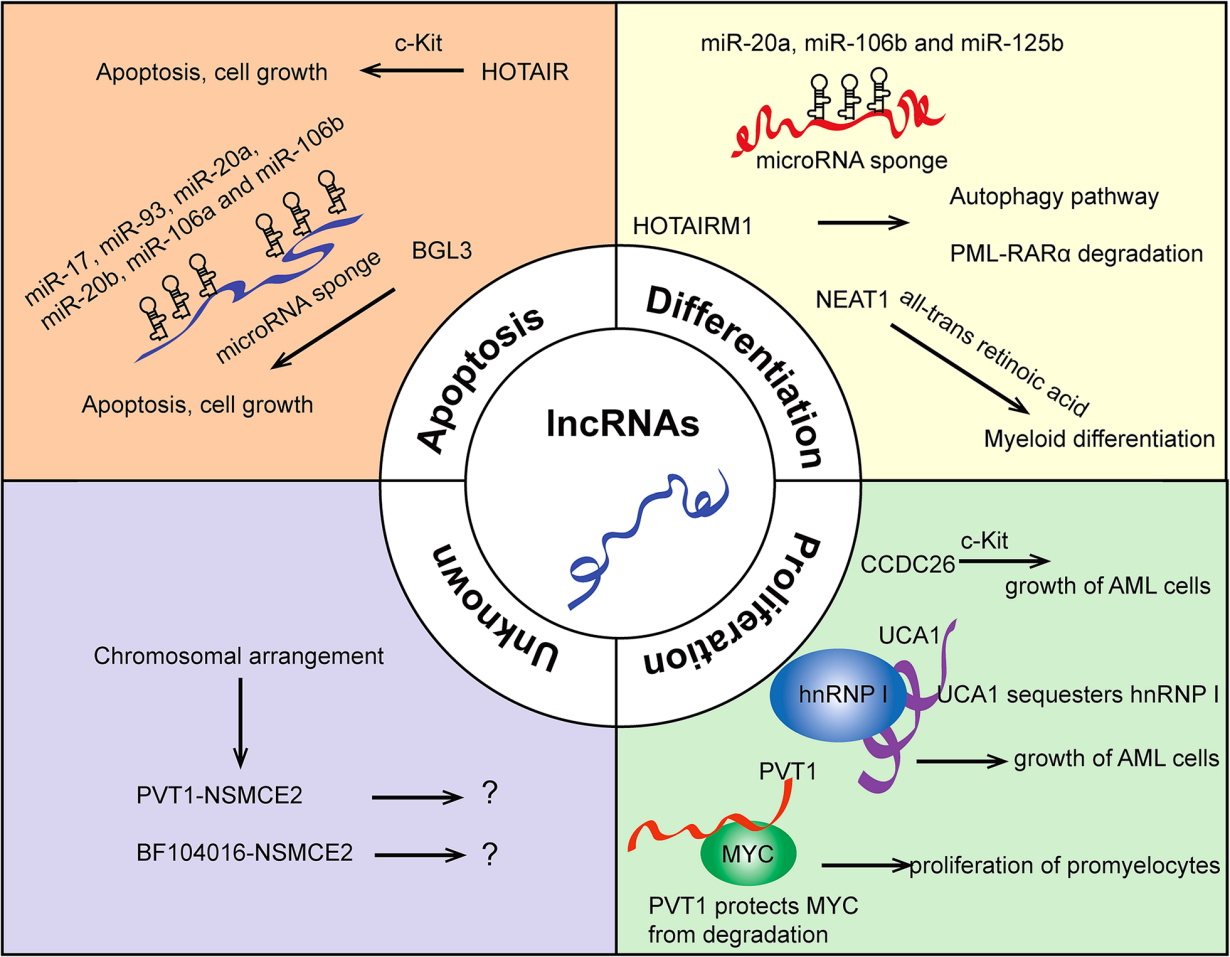
LncRNAs in acute myeloid leukemia. LncRNAs influence apopotosis, differentiation and proliferation of acute myeloid leukemia cells. Selected examples of AML lncRNAs and their molecular partners as well as the associated cellular phenotpyes are shown

LncRNAs exert their functions through multiple ways (Fig. 1).

First, lncRNAs can interact with other RNA molecules, e.g. microRNAs and mRNAs. The lncRNAs that can act by competing with endogenous RNA to sequester microRNAs include BGL3, CCAT-1, HOTAIR and HOTAIRM1. BGL3 functions as a competitive endogenous RNA for binding miR-17, miR-93, miR-20a, miR-20b, miR-106a and miR-106b to cross-regulate PTEN expression [24]. CCAT-1 exerts its oncogenic activity by sequestering tumor-suppressive miR-155, an inducer of apoptosis and cell differentiation, which is down-regulated in AML [25, 37]. HOTAIR competitively binds to and sequesters miR-193a, thus modulating the expression of c-KIT in AML cells [27]. A recent study showed that HOTAIRM1 regulates the ATRA-induced degradation of PML-RARα by acting as microRNA sponge sequestering miR-20a, miR-106b and miR-125b to target autophagy-associated genes [16]. The lncRNAs can also bind to the protein-coding transcripts and induce the translation inhibition. One such lncRNA is PU.1-AS. It is transcribed from the opposite strand of PU.1, a master transcription factor controlling myeloid differentiation. It has been found that PU.1-AS negatively regulates the expression of PU.1 by stalling the translation of PU.1 mRNA [19]. Furthermore, the preliminary data shows that PU.1-AS interferes with the binding of eEF1A to PU.1 mRNA, impairing the elongating complexes [19], although further studies are required to determine the precise mechanism.

Second, lncRNAs can bind protein partners to form a complex. For instance, a proposed mechanism for UCA1 action in proliferation regulation is to sequester hnRNP I, which is a positive translation regulator of p27 protein. Consistent with this hypothesis, it has been shown that UCA1 has a moderate binding capacity to hnRNP I and that UCA1 knock-down leads to an increased expression of p27 protein in AML cells [29]. Further evidence is required to support the hypothesis that UCA1 functions by sequestering hnRNP I, since the investigation of the biological effects and molecular mechanism underlying the interaction between lncRNAs and proteins is more difficult than the identification of factors interacting with the lncRNA of interest. A possible effect of the lncRNAs’ interaction with proteins is involved in regulating the stability of protein partners, as exemplified by PVT1. In both breast cancer and AML cells, it has been reported that PVT1 protects MYC from phosphorylation by the direct interaction with MYC, stabilizing and enhancing MYC [30].

Currently, orchestrating the chromosomal looping is a seldom-observed yet critical aspect regarding the action of lncRNAs in AML. Two lncRNAs have been reported to mediate the long-range regulation. Interestingly, both lncRNAs are transcribed from the loci that produce critical protein regulators. LncRNA RUNXOR is derived from the locus of transcription factor RUNX1 (AML1). It is 216 kb long and covers the entire locus of RUNX1, an important transcription factor in AML [13]. The 3′ UTR of RUNXOR is capable of binding to the promoter and enhancer of RUNX1, forming an intra-chromosomal looping modulating the expression of RUNX1. Also, RUNXOR participates in the long-range interaction between the chromosomes, and thereby might be involved in chromosomal translocations and leukemia development [13]. Another example is IRAIN, an imprinted lncRNA transcribed from the intron of insulin-like growth factor type I receptor (IGF1R), an oncogene promoting cell growth in AML cells. IRAIN interacts with chromatin DNA and participates in scaffolding the long-distance DNA regions to form an intra chromosomal loop involving the IGF1R promoter and the intronic enhancer [23].

However, apart from the cases mentioned above, the mechanisms of lncRNA action in AML are still largely unknown. Further studies are required to investigate the interactome of lncRNAs, and to determine the causal relationship between the direct physical interaction and the resultant phenotypic changes. For example, although HOTAIRM1 and NEAT1 are found to be required for ATRA-induced myeloid differentiation in AML cells, the mechanism of how they exactly exert their functions is poorly known.

## Regulation and dysregulation of lncRNAs in myelopoiesis

Emerging studies have indicated that lncRNAs specifically expressed during myelopoiesis are under the direct control of hematopoietic transcription factors. C/EBPα (CCAAT/enhancer-binding protein-α) is one of key regulators in myelopoiesis. A genome-wide study on investigating C/EBPα-regulated lncRNAs has revealed that 930 lncRNAs, including 600 up-regulated and 330 down-regulated, are regulated by C/EBPα [38]. At the individual gene level, a more in-depth study has shed light on the regulatory role of C/EBPα in the control of lncRNA UCA1 [29]. About 10% of AML cases bear CEBPA mutations [39], which leads to overexpression of C/EBPα-p30. Hughes et al. [29] and his colleagues identified that UCA1 is a novel target of both C/EBPα isoforms, i.e. C/EBPα-p42 and C/EBPα-p30, and the short isoform C/EBPα-p30 induces the expression of UCA1, leading to the abnormal up-regulation of UCA1 in CEBPA-mutated AML patients [29]. The work suggests the complexity of the transcriptional modulation of lncRNAs, as they could be controlled in a distinct regulatory pattern by different isoforms produced from the same gene.

PU.1 is another master regulator in myeloid differentiation. Most recently, we have found that the myeloid differentiation lncRNA HOTAIRM1 is a direct target of PU.1 [40]. The up-regulation of HOTAIRM1 during granulopoiesis depends on PU.1. Furthermore, low HOTAIRM1 expression is observed in APL cells, which is attributed to the reduced PU.1 expression, rather than the direct binding and repression by PML-RARα, the unique oncofusion protein in APL cells. This work identifies HOTAIRM1 as a novel target of PU.1, suggests the role of HOTAIRM1 in PU.1-mediated regulation network during myeloid differentiation and elucidates the mechanism by which HOTAIRM1 is deregulated in APL cells.

In addition to the direct regulation by transcription factors, lncRNA expression is closely associated with recurrent mutations in AML [39], indicating an indirect way of regulation. For example, lncRNA HOXB-AS3, MEIS1-AS2, PVT1, and CCD26 are up-regulated in cytogenetically normal AML patients with mutated NPM1. WT1-AS is associated with FLT3-ITD mutations. IDH1^R132^ mutated patient samples have up-regulated DLEU2 as well as down-regulated RP11-147N17.1. RUNX1-mutated patients have up-regulated vault RNA 1-1 (VTRNA1-1) [39]. Whether these observations merely represent a co-expression pattern or these lncRNAs indeed have roles in AML with the associated mutations remains to be explored.

Presently, mechanisms behind the dysregulation of lncRNAs in AML are far less clear than their biological functions. For example, previous studies have shown the involvement of PU.1-AS and EGO in myelopoiesis [19, 22], but whether they are dysregulated in AML is unknown, and how they are controlled during hematopoiesis and leukemogenesis is unexplored. Thus, more attention should be paid into the mechanisms behind the abnormal expression of lncRNAs in AML.

## Prognostic value and therapeutic promise of lncRNAs in acute myeloid leukemia

LncRNAs are associated with AML clinical features and outcomes. A recent study has shown that lncRNAs can be used to predict treatment response and outcome in older patients with cytogenetically normal AML. Garzon et al. have investigated the associations of lncRNA expression with clinical characteristics, recurrent mutations, and outcome in 148 cytogenetically normal older (age >60 years) AML patients and built a prognostic score based on the expression values of 48 lncRNAs that can been used to for outcome prediction [39]. Future studies are required to investigate whether these lncRNAs are functional in the AML development and whether they are regulated directly or indirectly by biologic changes influenced by specific mutations. Moreover, AML patients expressing a higher level of HOTAIR are associated with a worse clinical outcome in comparison with those with lower expression of HOTAIR [27]. Based on 215 intermediate-risk AML patients, Díaz-Beyá et al. [41] have reported that high HOTAIRM1 expression is independently associated with worse prognosis: a shorter overall survival and a higher cumulative incidence of relapse [41]. Besides, a higher HOTAIRM1 expression level is also associated with worse clinical outcome in AML patients with mutated NPM1 [41]. Overall, only a few published reports of lncRNAs’ prognostic value in AML are available currently, so more work is needed to explore the association between lncRNA and clinical characteristics, mutations and outcome.

LncRNAs are promising candidates for cancer therapy, especially considering some of them are tissue-specific drivers of cancer. Preclinical researches have implicated the efficacy of lncRNA-targeted therapeutics in certain types of cancer. In the MMTV-PyMT breast cancer mouse model, Malat1 exerts oncogenic activity by enhancing cell proliferation and tumor metastasis, and serves as a potential target for treatment [42]. Perturbation of Malat1 by RNA depletion induces differentiation of mammary tumors, increases cell adhesion and decreases metastasis [42]. However, to the best of our knowledge, there is still no therapeutic examples targeting lncRNAs in AML to date. Progress of developing lncRNA-targeted therapeutics is impeded by the limited understanding of their roles in AML and the molecular mechanisms by which they exert their functions in AML.

## Conclusions and perspectives

It becomes increasingly clear that lncRNAs play critical regulatory roles in AML. However, there are as yet unidentified lncRNAs and undiscovered machineries involved in AML. The road is still long before we fully understand their disease relevance and biological significance in AML. Genome-wide studies, in parallel with careful and in-depth investigations of individual lncRNAs, will greatly advance our understanding of how lncRNAs are involved in the initiation, maintenance and development of AML. Furthermore, the nature of the interactions between lncRNAs and other molecules, to a large extent, remains a mystery in AML. Resolving such issues requires the implementation of methodologies such as chromatin isolation by RNA purification (ChIRP) [43], capture hybridization analysis of RNA targets (CHART) [44] and RNA immune precipitation (RIP) to identify the binding chromatin DNA or binding proteins of lncRNAs. Finally, an imperative aspect of research is the inquiry at the mechanism under the dysregulation of lncRNAs in AML. In particular, for those functionally characterized lncRNAs that have regulatory roles in AML, it is really worthwhile to investigate how they are modulated in physiological conditions and whether they are dysregulated in the pathogenesis of AML. Understanding the mechanisms underlying lncRNA’ actions and dysregulation will ultimately pave the way to completely understand the pathology and to better treat AML patients.

## Abbreviations

lncRNA: long non-coding RNA
AML: acute myeloid leukemia
APL: acute promyelocytic leukemia
ATRA: all-*trans* retinoic acid
